# IS THE HEALTH OF OLDER AMERICANS WITH A GED EQUIVALENT TO THEIR PEERS WITH A HIGH SCHOOL DIPLOMA?

**DOI:** 10.1093/geroni/igac059.1255

**Published:** 2022-12-20

**Authors:** Esme Fuller-Thomson, Robin Grossman, Andie MacNeil

**Affiliations:** University of Toronto, Toronto, Ontario, Canada; University of Toronto, Toronto, Ontario, Canada; University of Toronto, Toronto, Ontario, Canada

## Abstract

Previous research has found higher levels of educational attainment to be strongly associated with better health outcomes in later life, such as better cognitive functioning and fewer functional and sensory impairments. However, most studies have grouped General Educational Development (GED) recipients with high school graduates, neglecting potential differences in socioeconomic status, health behaviours, and health outcomes among these two groups. The aim of the current study is to identify differences in the age-sex-race-poverty adjusted prevalence and odds of cognitive impairment, hearing impairment, vision impairment, limitations in activities of daily living (ADLs), and ambulation limitations among three groups of older American adults: high school dropouts, GED recipients, and high school graduates with no post-secondary education. The present study uses secondary analysis of the 2017 American Community Survey, a nationally representative survey of community-dwelling and institutionalized older adults aged 65 years and older, of whom 20,489 were GED recipients, 154,892 had a high school diploma and 49,912 had finished grade 8 but had not completed high school. Our findings indicate that there is a gradient in health outcomes among Americans aged 65-84, with the highest prevalence and odds of cognitive impairment, hearing impairment, vision impairment, ADL limitations, and ambulation limitations occurring among high school dropouts, followed by GED recipients, and the lowest prevalence among high school graduates. These findings suggestion that although GED recipients have better health outcomes than high school dropouts, there is still a significant disparity in health status between GED recipients and high school graduates.

